# Superoscillations without Sidebands: Power-Efficient Sub-Diffraction Imaging with Propagating Waves

**DOI:** 10.1038/srep08449

**Published:** 2015-02-13

**Authors:** Alex M. H. Wong, George V. Eleftheriades

**Affiliations:** 1 The Edward S. Rogers Sr. Department of Electrical and Computer Engineering, University of Toronto, 10 King's College Rd., Toronto, ON, M5S 3G4, Canada

## Abstract

A superoscillation wave is a special superposition of propagating electromagnetic (EM) waves which varies with sub-diffraction resolution inside a fixed region. This special property allows superoscillation waves to carry sub-diffraction details of an object into the far-field, and makes it an attractive candidate technology for super-resolution devices. However, the Shannon limit seemingly requires that superoscillations must exist alongside high-energy sidebands, which can impede its widespread application. In this work we show that, contrary to prior understanding, one can selectively synthesize a portion of a superoscillation wave and thereby remove its high-energy region. Moreover, we show that by removing the high-energy region of a superoscillation wave-based imaging device, one can increase its power efficiency by two orders of magnitude. We describe the concept behind this development, elucidate conditions under which this phenomenon occurs, then report fullwave simulations which demonstrate the successful, power-efficient generation of sub-wavelength focal spots from propagating waves.

Assuming ultimate resolution control on electromagnetic waves has been a long standing problem of significant scientific interest, with broad technological applications to imaging, lithography, sensing and therapy. While Abbé^1^, Rayleigh^2^ and others established that wave diffraction limits the controllability of an electromagnetic waveform to half its wavelength, recent works have shown that in special scenarios one can surpass this so-called diffraction limit to construct super-resolution devices. In particular, super-resolution devices based on evanescent waves^3^,^4^,^5^,^6^,^7^,^8^,^9^,^10^,^11^,^12^ (which for example include the Near-Field Scanning Optical Microscope) find great use in near-field electromagnetics and photonics. However, the fact that evanescent waves undergo exponential decay away from an object (or imaging device) restricts their working distance to less than half the operation wavelength. To achieve super-resolution at higher working distances, super-resolution microscopes have emerged which use non-linear optical effects^13^,^14^,^15^ or rely upon fluorescent labels^16^,^17^,^18^,^19^. While useful, these devices become specific to imaging certain classes of specimens or materials which prevents their widespread application.

The recent decade has seen several proposals of superoscillation imaging devices^20^,^21^,^22^,^23^,^24^,^25^,^26^, which have potential to become the method of choice for far-field general purpose sub-diffraction imaging. A superoscillation wave is a waveform which, across an arbitrary finite interval, oscillates faster than its constituent spectral components. This concept applies readily to super-resolution waveform control: using superoscillation theory, one can combine propagating electromagnetic waves to construct waveforms which contain spatial variations more rapid than the operation wavelength, and hence perform focusing or imaging with a resolution beyond the diffraction limit. However, with superoscillation waves one must be mindful of two limitations. First, superoscillation can only be achieved for a portion of the waveform. Second, previous work^27^ has shown that, as a direct consequence of the Shannon limit, superoscillation waveforms must feature high-energy sidebands on the image plane, outside the region of superoscillation. Energy within these sidebands varies exponentially with the superoscillation interval and polynomially with the reciprocal of the waveform bandwidth^27^. High-energy sidebands degrade the power efficiency, limit the field of view, and increase the required dynamic range of the imaging device. They also render the device very sensitive, as the high-energy portion of the waveform will likely scatter electromagnetic waves much more strongly than the sub-wavelength wave features within the region of superoscillation. Despite this limitation, sub-diffraction superoscillation hotspots have been demonstrated in both microwave and optical frequencies^23^,^28^,^29^, and techniques have been suggested to reshape the high-energy sidebands and separate them from the superoscillation hotspots^26^,^30^,^31^. Nevertheless, the existence of high-energy sidebands represents a major impediment to the realization of practical super-resolution systems based on superoscillation waves.

In this paper, we propose and demonstrate a method to altogether remove high-energy contents from a superoscillation waveform over an extended region of interest. Contrary to conclusions from previous works, we find that electromagnetic superoscillation waveforms can indeed be selectively generated in the absence of high-energy regions. In particular, we demonstrate through calculations and fullwave simulations how one can focus a series of propagating plane waves beyond the diffraction limit without generating high-energy spurious contents on the image plane. We also demonstrate that one can generate such a waveform with reasonable stability, such that high-energy regions are also avoided in a multi-wavelength region surrounding the image plane (in the longitudinal direction). This development paves way for the effective use of superoscillation super-resolution systems.

## Results

### Selective Superoscillation

We begin by proposing the concept of selective superoscillation. It is crucial to observe that previous works – which established rigorous relationships regulating the existence and energy ratio for superoscillation functions – focused on analyzing a superoscillation waveform either across an unlimited domain, or across one period. However, one can choose to synthesize a waveform over a selected interval which does not align with its period. By satisfying Maxwell's equations across a closed boundary, electromagnetic waveforms can be selectively excited across the confines of the cavity, and only within the cavity. This suggests it is possible to generate only the fast oscillation region of a superoscillation waveform without generating its high-energy contents. This paper numerically demonstrates this phenomenon.

### Superoscillation Waveform Design

**Figure 1:**
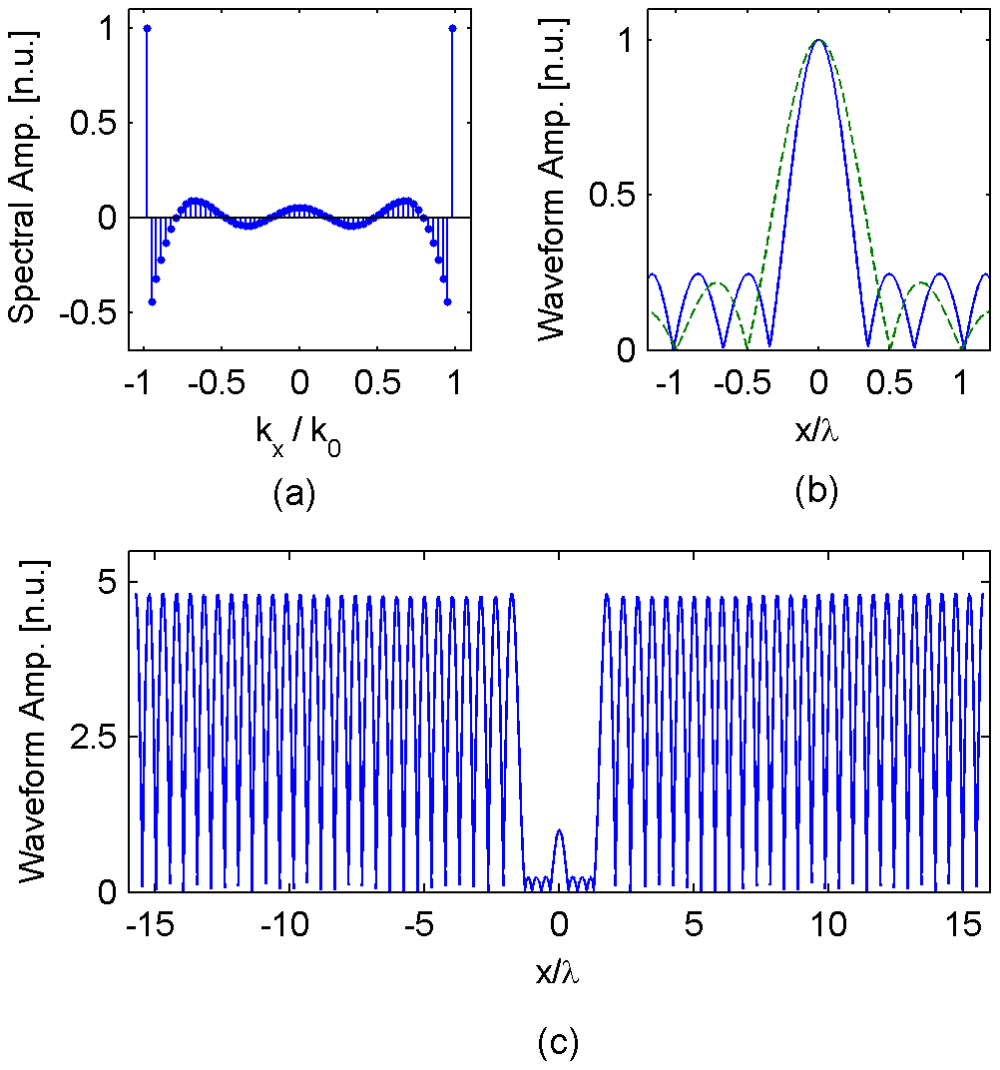
Superoscillation waveform design. (a) An amplitude plot of spectral (plane wave) components which make up the superoscillation waveform. Note that all plane waves are propagating, since for all of them |*k_x_*| ≤ *k*_0_. (b)–(c) The spatial profile of the superoscillation waveform, plotted (b) across the region of superoscillation (blue, solid), in comparison with the diffraction-limited sinc function (green, dashed) and (c) across one period of the waveform.

We design the desired superoscillation waveform through a procedure which draws inspiration from superdirective antenna theory. We refer the interested reader to the Methods section and our previous works^22^,^23^,^26^,^30^ which elaborate our design methodology. To crystalize the concepts proposed in this work, we consider a 2D environment in which plane waves propagate in the x- (transverse) and z- (longitudinal) directions. A superoscillation waveform will be constructed along the x-direction at the image plane *z* = 0, using *N* plane waves of a single operation frequency *f*, propagating in different directions to provide the needed bandwidth in the space of transverse spatial frequencies (*k_x_*-space). At the image plane, these plane waves come into focus to form the superoscillation wave. Figure 1 shows a design of interest with *N* = 63. Figure 1a shows the plane-wave amplitudes plotted against the transverse spatial frequency *k_x_*; Figs. 1b and 1c show the waveform amplitude within the region of superoscillation (Fig. 1b) and over one period (Fig. 1c). A plot of the diffraction-limited sinc function, whose spot width defines the Abbé diffraction limit, is plotted for comparison in Fig. 1b. The designed waveform is clearly sub-wavelength, with an electric field spot width (full width at half maximum, FWHM) of 0.42*λ*, which is 70% that of the diffraction limit.

### Superoscillation Waveform Propagation

Away from the image plane, the superoscillation waveform drifts out of focus due to diffraction. We employ the plane-wave decomposition method to calculate the waveform across the xz-plane. First, we find the corresponding longitudinal spatial frequency (*k_z_*) for each plane wave through the dispersion relationship
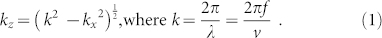
Here *k*, *λ* and *v* represent respectively the wavenumber, wavelength and wave velocity in a simple electromagnetic medium, at the operation frequency *f*. Thereafter, the corresponding electric and magnetic field phasors can be found by
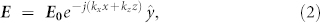



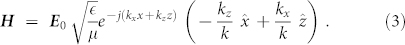
Here 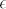 and *μ* represent the permittivity and permeability of the medium and ***E*_0_** is the complex amplitude of the electric-field phasor. An *e^j^*^2*πft*^ time-dependence is assumed.

Invoking the equivalence principle of electromagnetics, we can selectively generate each plane wave within a closed region by providing electric and magnetic current excitations across the region boundary^32^. These currents are related to the co-located electromagnetic fields through
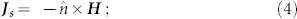



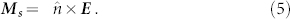
where ***J_s_*** and ***M_s_*** represent respectively the electric and magnetic currents at the boundary surface and 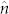 is the outward-pointing normal. Superimposing required currents for each plane wave, according to predesigned weights shown in Fig. 1a, synthesizes the target superoscillation within a selective region of interest, which excludes high-energy regions at the image plane.

### Numerical Simulation

**Figure 2:**
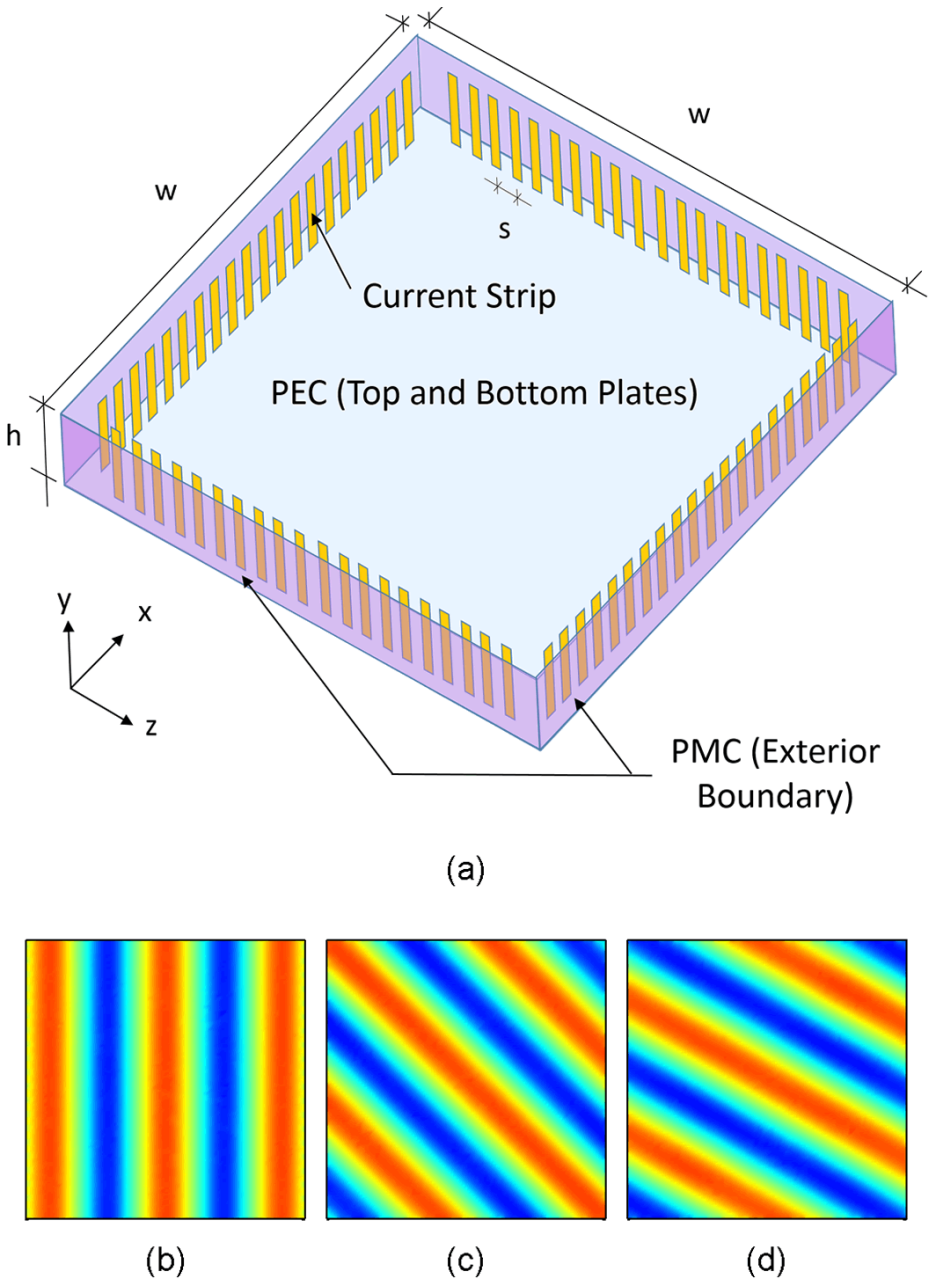
Selective superoscillation generation. (a) A schematic of the selective superoscillation generation device. Key dimension are as follows: *s* = 0.12*λ*, *w* = 2.4*λ* and *h* = 0.05*λ*. (b)–(d) The instantaneous electric field generated for three plane waves, propagating at (b) 0°, (c) 45° and (d) 60°, with respect to the +z-axis. In all three cases 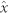 points upward while 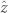 points to the right.

To validate the concept of selective superoscillation, we perform fullwave simulation using Ansys HFSS – a commercial software which solves Maxwell's equations using the Finite Element Method. Figure 2a shows a schematic of the simulation. The simulation resides within a parallel-plate waveguide with sub-wavelength separation. This renders y-direction invariant and constrains the study to the x- (transverse) and z- (longitudinal) directions in accordance to our designed superoscillation waveform. In this environment, the electric field points in the y-direction, while the magnetic field points in the x- and z- directions. Applying equations (4) and (5) along the boundary results in a set of y-directed electric current and x- and z-directed magnetic current. We generate the required electric current by placing sub-wavelength current strips, spaced *s* = 0.12*λ* apart (center-to-center), along the boundary of a square cavity of width *w* = 2.4*λ*. Instead of generating the required magnetic currents, we place a perfect magnetic conductor (PMC) directly behind the electric current strips. The PMC boundary causes an equivalent effect as the required magnetic currents do to the electromagnetic field within the cavity, in accordance to Love's equivalence principle^32^. Figure 2b–d plots the instantaneous electric field for synthesized plane waves propagating at 0°, 45° and 60° with respect to the longitudinal (z) axis. As observed, individual plane waves are faithfully synthesized within the computation environment.

**Figure 3:**
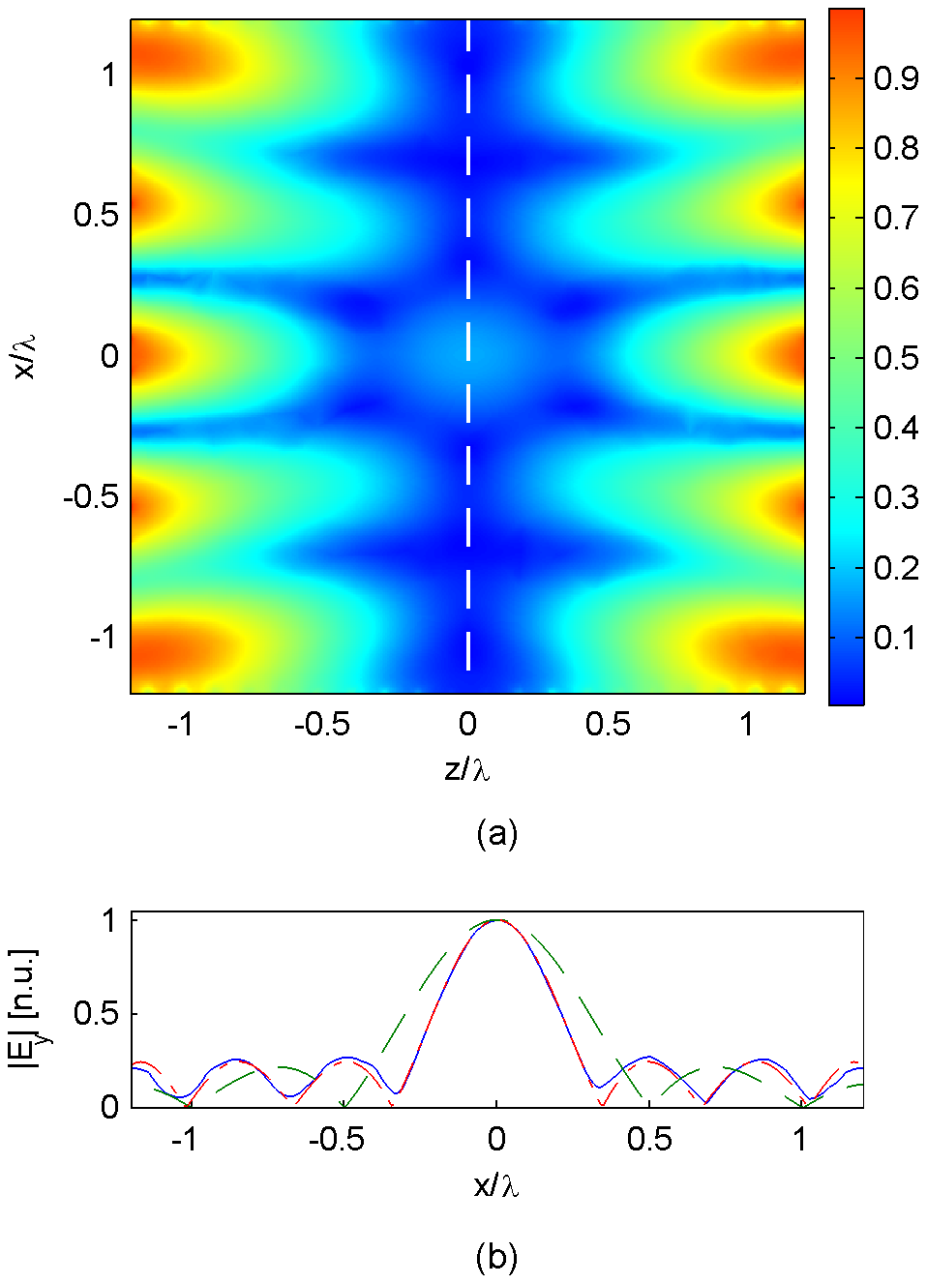
Numerical simulation results for close cavity selective superoscillation generation. (a) The time-averaged electric field amplitude across the field generation domain. The white dashed line denotes the image plane. (b) The simulated waveform along the image plane *z* = 0 (blue, solid), plotted against the design waveform (red, dash-dot) and the diffraction-limited sinc function (green, dashed).

Synthesizing the superposition of plane waves shown in Fig. 1 results in the electric field distribution shown in Fig. 3. Figure 3a plots the time-averaged electric field across the cavity; Fig. 3b plots the electric field across the image plane (*z* = 0) alongside the designed field profile and the diffraction-limited sinc function. Comparison with the calculated field profile shows excellent agreement within the region of selective synthesis; comparison with the diffraction-limited sinc function shows a sub-wavelength focus: the synthesized electric field has a focal width (at half maximum) of 0.42*λ*, which is 70% that of the diffraction-limited sinc function.

Figure 3a also shows the electric field profile away from the image plane. We observe that the waveform retains a sub-wavelength focus along the x-direction over a short longitudinal range, from *z* = −0.2*λ* to *z* = 0.2*λ*. However, the sidelobe level increases as we move away from the prescribed image plane. A diffraction-limited focal pattern is also formed across the principal (z) axis. In addition, the waveform amplitude has been kept low within the synthesis area with the exception of a few hotspots near the edge of the cavity. The absence of high field regions suggests that the superoscillation waveform can be synthesized in a power efficient manner. We will elaborate along this front in the Discussion section of this paper.

### Open Cavity Simulation

We further investigate the possibility of generating similar waveforms in an open environment. By choosing the positive root for *k_z_* in equation (1) we have chosen to design our superoscillation waveform with plane waves which travel in the +z-direction. Since a close approximation of these plane waves can be produced by reproducing the appropriate electric and magnetic currents for the region *z* ≤ 0, we hypothesize that a sub-wavelength superoscillation waveform can be produced (without high-energy components) with a semi-open cavity, which only includes excitation currents from *z* ≤ 0. We performed the corresponding simulation to verify this hypothesis. Our open cavity simulation follows the setup shown in Fig. 2a, with a few modifications. Firstly, we invoke a change in co-ordinates by defining *u* and *v*,
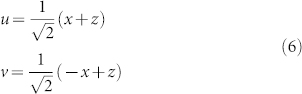
Under this transformation, all plane waves are effectively rotated by 45° upon a mapping from the xz-plane to the uv-plane. The principal (longitudinal) axis now lies on the line *y* = 0; *u* − *v* = 0 while the image plane lies on *u* + *v* = 0. Secondly, we retain the same cavity boundary in the uv-plane as it was in the xz-plane (i.e. it forms a square, not a rhombus, in the uv-plane). However, we remove current excitations and the PMC boundary at the +u and +v-directions, and replace them with a radiation boundary. Finally, small adjustments are made to the plane wave coefficients to optimize sidelobe performance at the image plane.

**Figure 4:**
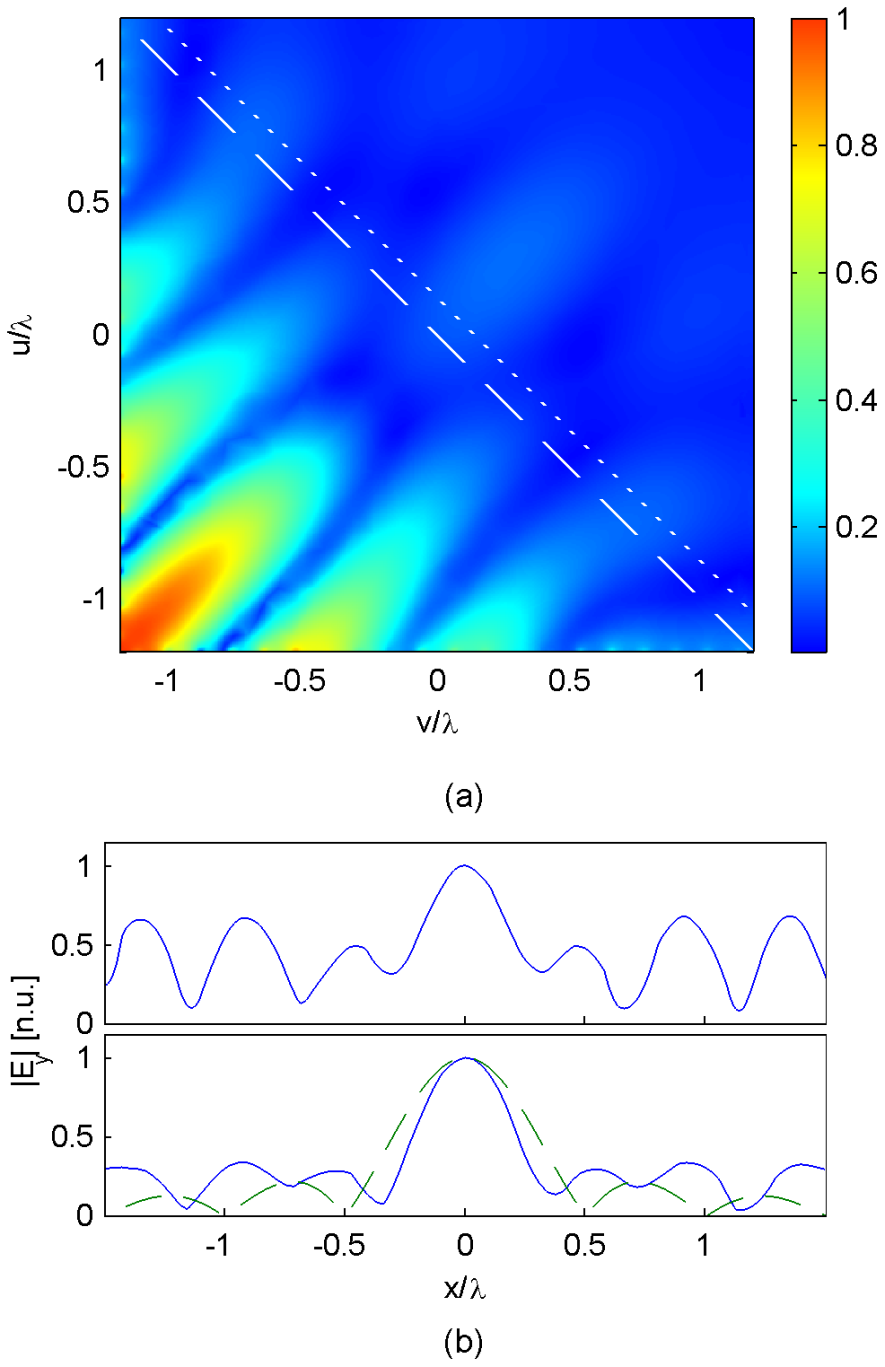
Numerical simulation results for open cavity selective superoscillation generation. (a) The time-averaged electric field amplitude across the computation domain. The white dashed line denotes the image plane; the white dotted line denotes the modified image plane. (b) The simulated waveform along the image plane *u* + *v* = 0. (c) The simulated waveform along the modified image plane *u* + *v* = 0.2*λ* (blue, solid), plotted alongside the diffraction-limited sinc function (green, dashed).

Figure 4 shows the results of this simulation. Figure 4a shows the time-averaged electric field amplitude within the computation domain. Clearly resemblance to Fig. 3a is achieved in the region *u* + *v* < 0, where the excitation currents remain intact. However, the waveform deviates from that shown in Fig. 3a in the half-space *u* + *v* > 0, since the generation of plane-wave components is incomplete without having excited the relevant sources in that half-space. At the original image plane *u* + *v* = 0 (Fig. 4b), one continues to see a sub-wavelength peak, but accompanying sidelobes have become of appreciable strength. Notwithstanding, at a nearby plane of *u* + *v* = 0.2*λ* (Fig. 4c), a sub-wavelength peak of focal width 0.44*λ* can be observed, amidst subdued sidelobes similar to the level of Fig. 3b. This focal width is 0.44*λ*, or 73% that of the diffraction limit sinc – which has also been plotted in Fig. 4c for comparison.

## Discussion

We have demonstrated that sub-wavelength superoscillation waveforms, without accompanying high-energy regions, can be generated in close cavity and open cavity environments. Fullwave numerical simulations show the formation of sub-wavelength hotspots from propagating plane waves, with dimensions which are 70% (close cavity) and 73% (open cavity) of the diffraction limit, respectively. In both demonstrations, high-energy regions are largely avoided – both on the image plane and within the selective region for waveform synthesis. The mitigation of high-energy regions also served to reduce tenfold the current required to synthesize the waveform. More aggressive superoscillation waveforms can be constructed to achieve tighter focusing on the image plane, albeit at the likely cost of enhancing hotspots away from the image plane. The synthesis of a sub-diffraction superoscillatory focus, removed from its high-energy sideband, by sources more than one wavelength away, removes a major impediment towards the practical realization of superoscillation-based super-resolution systems.

Our next remark contrasts the selective superoscillation cavities proposed in this work with evanescent-wave-based imaging devices. Firstly, while our proposed selective superoscillation cavities achieved sub-wavelength focusing in the near-field, the demonstrated cavity size of 2.4 wavelengths gives much larger operation room compared to evanescent-wave-based imaging devices, for which the image typically lies within a quarter-wavelength from the device. A larger image distance is achievable with more aggressive superoscillatory function designs. Secondly, in contrast to an exponential field decay which takes place between the device and the image plane in typical evanescent-wave-based focusing devices, no such field decay takes place in selective superoscillation devices. This is because the waveform is composed of propagating plane waves, as opposed to evanescent plane waves. As we have earlier observed, the selective superoscillation cavities reported in this work achieved electric-field focusing in both lateral and longitudinal directions. The fact that one can escape exponential field decay (and even achieve focusing) in the longitudinal direction enables selective superoscillation devices to achieve longer image distances, and improves prospects for its practical implementations.

On the other hand, for the open-cavity device, the image distance is in some sense compromised, since the adjusted image plane (the dotted line in Fig. 4) – which is now in open space – is only a fraction of a wavelength away from the two ends of the excitation surface: (*u*, *v*) = (1.2*λ*, 0) and (*u*, *v*) = (0,1.2*λ*). This is inevitable, since the absence of sources and discontinuities in the half-space *z* > 0 means that at *z* = 0^+^ the field profile can once again be decomposed into plane waves and evanescent waves across a sourceless space, and in this process sub-diffraction details of the waveform (which are unaccompanied by superoscillation sidebands) will be decomposed into evanescent waves which decay in the z-direction. Hence, one can view the open cavity selective superoscillation device as a specialized evanescent-wave-based focusing device, with an exit facet at *z* = 0. Notwithstanding the open cavity case demonstrated three novel features: (i) a sizeable enclave of the cavity, (ii) a focal peak which stood at least 1.2 wavelengths away from any part of the device, and (iii) the absence of exponential field decay from the device to the focal spot. These features combine to present practical advantages for object placement and scanning, which have been hurdles for conventional evanescent-wave-based imaging devices.

In addition to observing the field distribution, we investigated the power efficiency of the proposed selective superoscillation scheme. We calculated the total current required to generate the waveform shown in Fig. 2a and compared it to the total excitation current in a scenario considered in Ref. 23 – where an array of current sources are placed along a source plane to generate the superoscillation waveform (with its sidebands) at an image plane five wavelengths away from the source. The comparison shows that the (close cavity) selective generation method hereby proposed reduces the total current by a factor of 10. The open cavity method further reduces current consumption by half, though at an expense of slightly reducing the focal strength at the modified image plane. Hence, a selectively generated superoscillation waveform is doubly beneficial: it avoids generating the high-energy region and in doing so it dramatically lowers the energy cost in signal generation. This combination of advantages makes selective superoscillation potentially suitable for efficient sub-wavelength wireless microwave and/or optical power transfer – with important applications in lithography, healthcare and microwave thermotherapy.

Lastly we remark on the practicality of building a selective superoscillation device. While in this work, we demonstrated the selective synthesis of a superoscillation waveform with utmost simplicity by utilizing current sheets backed by perfect magnetic conductors (PMCs), we note that electromagnetically equivalent boundary conditions can also be generated using current loops backed by perfect electric conductors (PECs), or a combination of current strips and loops. Metasurfaces with such elements have recently been demonstrated^33^,^34^,^35^. An experimental demonstration of selective superoscillation synthesis is hence within reach and will be the subject of forthcoming work. We expect that selective superoscillation devices will pave the way to highly novel and practical super-resolution systems with wide ranging applications from microwaves to optics, especially in imaging, lithography, sensing and therapy.

## Methods

### Superoscillation function design

Our design procedure for 1D superoscillation functions draws inspiration from superdirective antenna design. We consider a periodic function *F*(*x*) with Bloch period Λ, which is composed of *N* complex exponentials
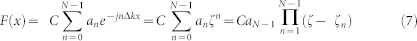
where 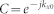 and *ζ* = *e*^−*j*Δ*kx*^. Here *k_x_*_0_ and Δ*k* = 2*π*/Λ respectively represent the component with the lowest (most negative) spatial frequency and the spacing between adjacent spatial frequency components. The factorization of *F*(*ζ*) (*F*(*x*) written as a function of ζ) allows us to visually represent it as a function with *N* − 1 zeros in the complex ζ-plane. On this complex ζ-plane, *F*(*x*) traces a path along the unit circle, traversing once every time x increases by one Bloch period Λ. Thus choosing the zeros of the complex function *F*(*ζ*) represents an intuitive way to design the function *F*(*x*).

In earlier works, we have established that to construct a superoscillation function, one needs to choose zeros for *F*(*ζ*) in the same way as if one would design a superdirective antenna: namely, we close-pack a portion of the available zeros along a section of the unit circle, and hence arrive at a waveform *F*(*x*) which super-oscillates along that interval. While the energy content of *F*(*x*) inevitably increases outside the superoscillation region, in this paper we have shown that one can mitigate this ill effect by excluding the high-energy region from the selective generation area.

In our design we used *N* = 63 plane waves, evenly spaced in the transverse spatial frequency (*k_x_*) domain between ±*k*_0_ = ±2*π*/*λ*, where the wavelength is *λ* = 100 mm for an operation frequency *f* = 3 GHz. We place 8 zeros within a superoscillation region |*x*| < 1.3*λ* through a Tschebyscheff expansion algorithm to form the narrowest possible peak, and equi-ripple sidelobes with field strength 25% that of the peak. After this initial placement of zeros, we place the remaining zeros to minimize the sideband amplitude. Even though the high-energy regions would eventually be excluded, we find that somewhat reducing the sideband amplitude in the function design process improves the stability of the superoscillation waveform, and enables one to also avoid high-energy regions in close longitudinal proximity to the image plane.

Figure 1 shows the results of the design, displaying the plane waves used and a spatial profile of the waveform. Figure 1b clearly shows the designed waveform forms a focus beyond the diffraction limit.

## Author Contributions

The authors jointly conceived and refined the underlying concept to the work. A.M.H.W. developed the theory, and performed calculations and simulations. G.V.E. provided guidance to the project. Both authors contributed to the manuscript.
